# Automation of dissolution tests

**DOI:** 10.1155/S1463924603000026

**Published:** 2003

**Authors:** Rolf Rolli

**Affiliations:** SOTAX AG Basel Binningerstrasse 106 Allschwil CH-4123 Switzerland

## Abstract

Dissolution testing of drug formulations was introduced in the 1960s and accepted by health regulatory authorities in the 1970s. Since then, the importance of dissolution has grown rapidly as have the number of tests and demands in quality-control laboratories. Recent research works lead to the development of in-vitro dissolution tests as replacements for human and animal bioequivalence studies. For many years, a lot of time and effort has been invested in automation of dissolution tests. There have been a number of in-house solutions from pharmaceutical companies and many have created task forces or even departments to develop automation. Robotic solutions with sequential operation were introduced as well as the simultaneous operation concept developed by SOTAX. Today, pharmaceutical companies focus their resources mainly on the core business and in-house engineering solutions that are very difficult to justify. Therefore, it is important to know the basic considerations in order to plan an automation concept and implement it together with a vendor.


Journal of Automated Methods & Management in Chemistry
Vol. 25, No. 1 (January–February 2003) pp. 7–15

Taylor & Francis

Taylor & Francis Group

### Automation of dissolution tests

**Rolf Rolli**

*SOTAX AG Basel, Binningerstrasse 106, CH-4123 Allschwil, Switzerland. e-mail: rolf.rolli@sotax.ch*

*Dissolution testing of drug formulations was introduced in the 1960s and accepted by health regulatory authorities in the 1970s. Since then, the importance of dissolution has grown rapidly as have the number of tests and demands in quality-control laboratories. Recent research works lead to the development of in-vitro dissolution tests as replacements for human and animal bioequivalence studies. For many years, a lot of time and effort has been invested in automation of dissolution tests. There have been a number of in-house solutions from pharmaceutical companies and many have created task forces or even departments to develop automation. Robotic solutions with sequential operation were introduced as well as the simultaneous operation concept developed by SOTAX. Today, pharmaceutical companies focus their resources mainly on the core business and in-house engineering solutions that are very difficult to justify. Therefore, it is important to know the basic considerations in order to plan an automation concept and implement it together with a vendor.*

### Introduction

Laboratories automate dissolution tests to increase capacity, improve accuracy and reduce costs per test. These factors lead one to consider automation as a method of choice for a quality-control laboratory as well as for a research laboratory. In addition, the emerge of service laboratories testing samples outsourced from pharmaceutical companies calls for automation to offer clients an economic service.

Increasing capacity is a result of a general increase in a number of tests due to the following factors.

- Obligation to perform dissolution tests on older drugs.
- Increased demand in the number of stability tests.
- Higher demand on tests per production batch.
- SUPAC changes.
- *In-vitro* dissolution test replacing bioequivalence studies.
- Higher accuracy as in manually performed test leads to high variances in results.

In addition, an increasing number of modified release drugs require dissolution tests running for several hours. Often, these tests have sampling points overnight or even over weekends. Increasing the test length leads to a considerable reduction in test capacity. The needed higher capacity can be created by adding more test instruments and hiring additional laboratory personnel. A more economic way is to use non-used night and weekend times with automated systems.

Specifications for release rates are getting narrower. Manual tests with different personnel often create considerable differences in results, resulting in high costs as production batches cannot be released or have to be re-analysed. Out-of-specification results requiring additional testing and the preparing of reports to explain the variances have become a main issue for quality-control laboratories. Therefore, automation concepts offer the advantage of reproducible results and all required documentation including calculations, statistical data and graphs on the press of the print button. The introduction of CFR 21 Part 11 and the request of the US Food and Drug Administration (FDA) for electronic records are more reasons for opting for automated laboratory tests.

The documentation required for quality control tests is growing. Consequently, the output per laboratory technician on average is four short time tests (30 min) per day during a normal working day (8 h). This results in only 800 tests per person-year. The output could be even lower owing to the introduction of the 35-h working week in some countries and a further increase in documentation requirements. This would increase the cost per dissolution test even further as additional manual dissolution baths and staff would have to be added. To maintain or lower the cost per test, an investment in automation is the first choice.

Automation offers not only benefits, but also challenges, which the manufacturer has to overcome:

- easy validation of hardware and software;
- robust systems that can be used 24 h a day in routine;
- efficient sampling system;
- efficient cleaning system;
- choice of media change procedures;
- high accuracy and reproducibility;
- flexible concepts allowing customized solutions; and
- high throughput for maximal productivity increase for the customers.

SOTAX has been involved in the field of dissolution for many years and has developed automation concepts for most pharmaceutical laboratories, often creating sophisticated customized solutions that solve specific needs. We differentiate between the automation of a single test, a semi-automated dissolution system and the automation of a series of tests, a fully automated dissolution system.

In a breakdown of a dissolution test procedure into single steps, the differences between semi- and fully automatic systems can be summarized as given in table 1.

If a laboratory intends to automate dissolution tests, it has to analyse its annual requirements first and consider the following criteria:

- quantity of tests;
- type of test (USP 1/2/4/5/6);
- duration of tests;
- pH changes during the test;
- media replacement: yes or no;

*Table 1. Example of the paddle method, USP 2.*

| *Test step* | *Semi-automatic system* | *Fully automatic system* |
|---|---|---|
| Medium preparation | manually | automatic |
| Medium degassing | manually | automatic |
| Start of the method | automatic | automatic |
| Tablet dropping | automatic | automatic |
| Sampling | automatic | automatic |
| Standard monitoring | automatic | automatic |
| Medium replacement | not possible | possible |
| pH change | limited possibility | possible |
| Medium change | not possible | possible |
| Calculation | automatic | automatic |
| Emptying of vessels | manually | automatic |
| Cleaning of the system | manually | automatic |
| Start of the next test | manually | automatic |

- time and number of sampling points;
- standard monitoring: yes or no; and
- how many different medias are used.

There are different options in the automation of the USP 1 and 2 methods that differ significantly from automation of the USP 5/6 methods. USP 4 methods differ even more in the equipment concept from the other methods. For USP 3, there is currently no real automation available; therefore, it is not covered here.

### Automation of the USP 1 and 2 tests

One of the most laborious operations in a dissolution test is the medium preparation. Some companies have outsourced medium preparation to external companies and use prefabricated medium supplied plastic bags. This reduces the required time considerably and adds to accuracy as the medium is produced in large quantities, is analysed, and its shelf life tested and supplied with a CoA (certificate of analysis). While the advantages are obvious, the logistic problems are considerable with transport costs and the storage of the medium. A dissolution test requires about 6–7 litres of medium and 10 tests a day are common, even for a small laboratory. This would require 350 litres of medium per week.

A better option is a medium preparation device, which is the first step in automation. Different devices are available from several vendors. Some are simple desktop models with limited volume and features, others are movable but rather large. The SOTAX Medium Preparation Station (MPS) is compact with the following features (see figure 1).

- Mobile and space-saving unit.
- Open system, easy to clean, observe and validate.
- Exact preheating of the medium to the test temperature and reliable degassing of the dissolution medium.
- Continuous maintenance of the selected temperature.
- Fast and accurate volumetric filling of test vessels at 2000 ml/min.

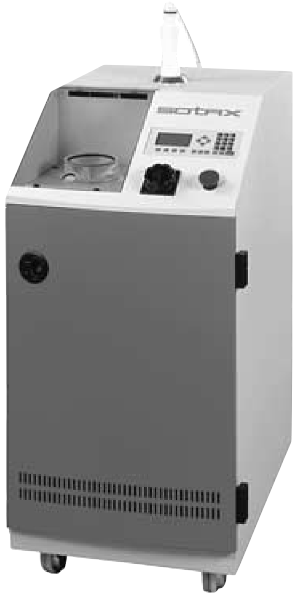

*Figure 1. Medium Preparation Station (MPS).*

- Use of concentrates with online preparation or by sequential addition.
- Option of gravimetric self-calibration with built-in balance and automatic report generation.

### Semi-automated systems

For a high number of samples per year, a fully automated test instrument is the most promising solution as 2000 batches or more per year can be tested with a limited number of personnel. Otherwise, semi-automated solutions are of great importance when only single tests have to be automated. Semi-automated systems by SOTAX are based on a modular concept and can be customized. There are off- and online solutions or a combination of these types available.

In the application of offline systems, the dissolution test and subsequent monitoring of the samples are separated. This solution is very beneficial if shared equipment like spectrophotometers or high-performance liquid chromatographs (HPLCs) are used to analyse the samples.

With offline systems there are different formats of racks available. One kind is for HPLC vials. Another kind is for larger tubes for dilution steps or chemical modifications; this sample can be analysed by an ultraviolet (UV) or HPLC method. As with this method, an aliquot of the test volume is taken out, the media replaced or mathematical compensation is required. With an AT 7*smart* system, the collected volume can be replaced automatically. This is of considerable importance when testing drugs under already unfavourable sink conditions. With this hardware configuration, any volume correction in the calculation is no longer needed.

There are also different online system options available that measure samples directly. The configuration depends very much on the applied analytical method. We distinguish between the following types:

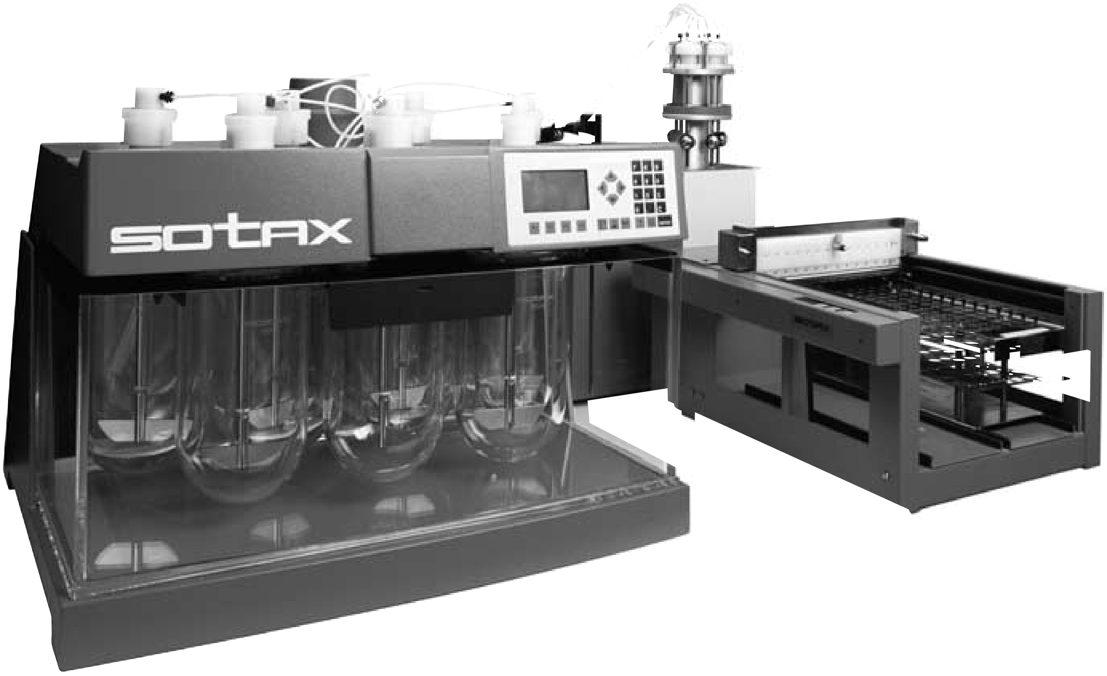

*Figure 2. AT 7*smart *Off-line with the Fraction Collector.*

- UV online systems with a spectrophotometer;
- UV online systems with fibre-optic probes;
- HPLC online systems; and
- combined system on-/offline.

UV online systems with a spectrophotometer are very widely used and are reliable semi-automated solutions. WinSOTAX, the dissolution software described below, provides software drivers for most used types of spectrophotometers including those from Perkin Elmer, Agilent, Shimdazu, Unicam and Beckman. UV online systems are widely used as they represent the highest security and comfort level for laboratories.

With the modular concept, many other hardware configurations like double systems are possible for S2/S3 testing or bioequivalence studies. In addition, systems allowing pH changes with a media selector for up to three media are possible. This allows one to perform *in-vitro/in-vivo* studies or tests for extended release forms. Additional options like solvent replacement after sampling and solvent addition for pH change are available. In addition, the on-/offline option is very popular as it gives the laboratory flexibility either to measure online or to collect a sample, or both. This may be used for multi-component drugs where one active substance is measured by means of UV and the other by HPLC. In addition, in UV measurement a collection of samples is popular as this retainer sample can be tested in case of an unexpected result. In this case, only the collected sample is retested and for investigation the test does not have to be repeated.

HPLC has replaced UV detection in many cases although the cost for equipment and reagents is much higher. HPLC and dissolution testing do not match very well, as dissolution tests results in a high number of samples, which the HPLC system cannot analyse in the same time as it is a rather slow method despite considerable improvement by manufacturers. Sample buffer storage must be used and fraction collection still remains for most laboratories the optimal solution, but HPLC online has its niche application with longer test times with one or two sample points. HPLC online can also be used for dissolution tests overnight and at weekends. A hardware solution is available which is connected to most commercial available HPLCs. The AT 7*smart* HPLC online system uses five rows of seven flow cuvettes that store the samples. The system cleans a row after the last sample is tested by the HPLC system and is again available for another sample point. WinSOTAX controls the sampling, dilution and injection of samples and standards.

### Fully automated systems

With this system type, laboratories achieve the highest productivity gain as the system is loaded with multiple batches of samples and performs test after test with all necessary steps being automated. There are, however, a number of issues to consider.

- Easy validation of hardware.
- CFR 21 Part 11 software-controlled system.
- Mechanically robust systems to be used 24 h a day in routine.
- Efficient media preparation and dispensing.
- Accurate sampling system.
- Efficient cleaning system to eliminate carry over.
- Choice of media change procedures.
- Very high accuracy and reproducibility.
- Flexible concept to allow customized solutions.
- Very high throughput to give maximum productivity increase to customers.

There are different system types like robotic systems with moving arms, up grades of regular baths with sampling, medium feed and cleaning devices on the market. SOTAX has a different concept and offers since a few

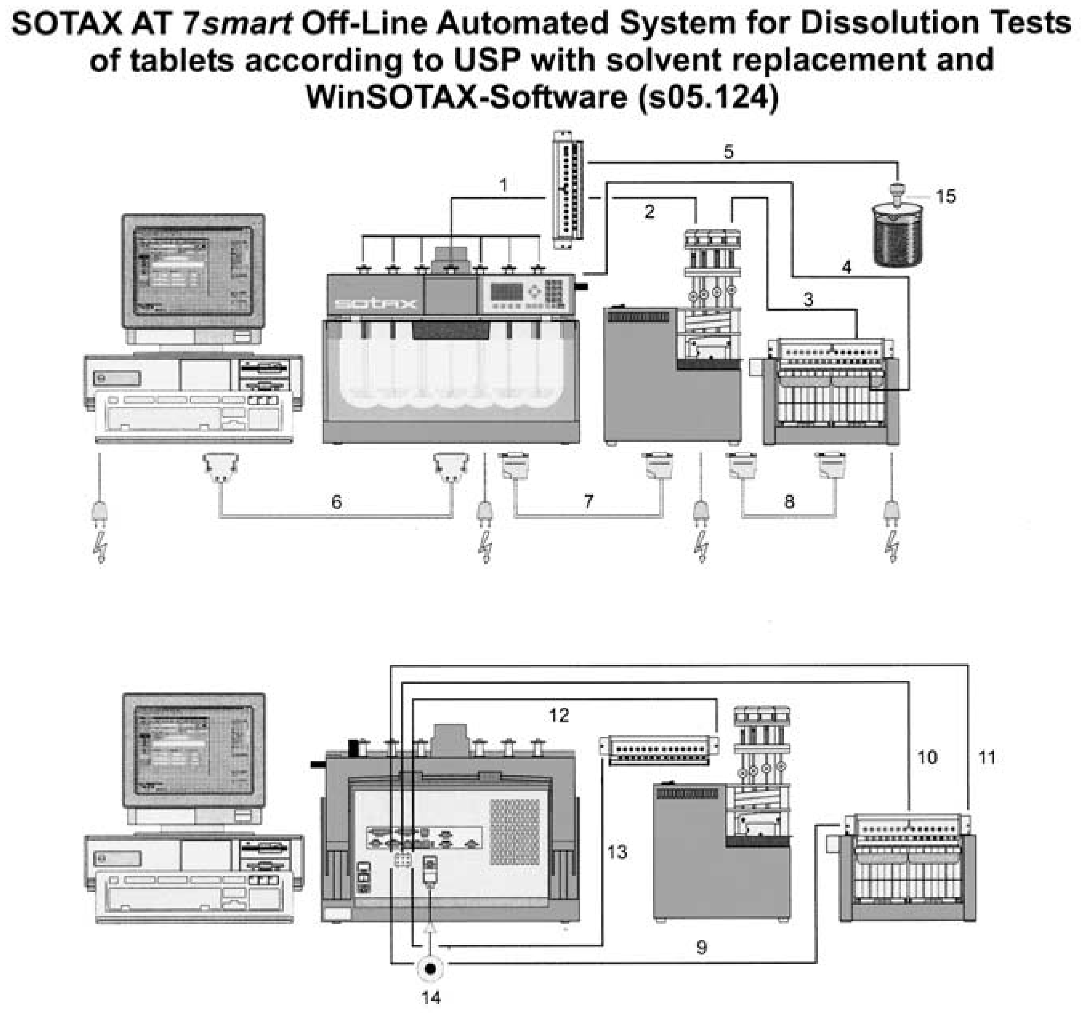

| No. | Designation | Art. No. |
|---|---|---|
| 1 | Bundle-Tubes 7 x 1.5/3 x 1300 mm | 4162-1 |
| 2 | Bundle-Tubes 7 x 1.5/3 x 780 mm | 4161-1 |
| 3 | Bundle-Tubes 7 x 1.5/3 x 780 mm | 4161-1 |
| 4 | Return-Line | 7677 |
| 5 | Bundle-Tubes 7 x 1.5/3 x 1300 mm | 4161-2 |
| 6 | Control cable RS-232 / 3 m | C100-0176 |
| 7 | Control cable 9 x 1.6 m | 3483-2 |
| 8 | Control cable 9 x 0.6 m | 3483-1 |
| 9 | Tube Black 2/4 x 2700 | R930-9010 |
| 10 | Tube Blue 2/4 x 2700 | R930-9012 |
| 11 | Tube White 2/4 x 2700 | R930-9020 |
| 12 | Tube Red 2/4 x 2000 | R930-9014 |
| 13 | Tube Green 2/4 x 2000 | R930-9016 |
| 14 | Air Tube 6/4 mm | R745-9090 |
| 15 | Suction part with filter | 6273 |

*Figure 3. AT 7*smart *Off-line System with Solvent Replacement.*

years the third-generation of fully automated system, the SOTAX AT 70*smart*. High numbers of this type of system are installed in R&D, quality control and stability testing laboratories in Europe and the USA. Installation sites include the laboratories of big multinational companies, of generic manufacturers, of contracting companies as well as of health authorities. The SOTAX AT 70*smart* is a fully integrated automation system managing all operations in a total of seven vessels simultaneously. This results in enormous timesavings. With this system, up to 15 USP 2 tests are fully automated, from the tablet input up to the print out of the report. The main features are as follows.

- Mechanically very robust to be used 24 h a day in routine.

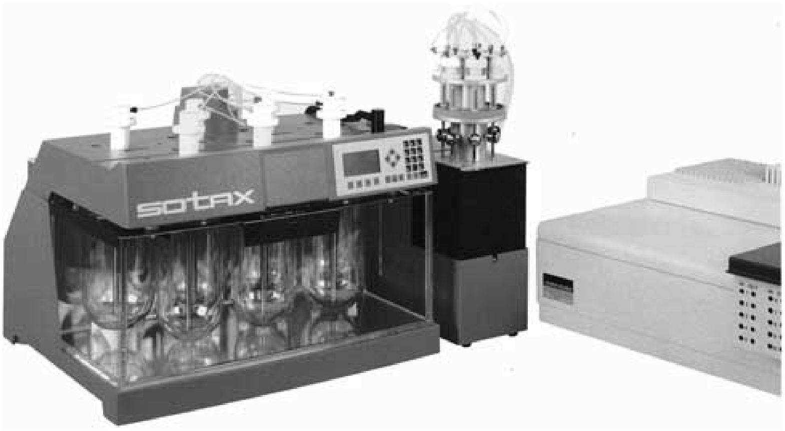

*Figure 4. AT 7*smart *On-line.*

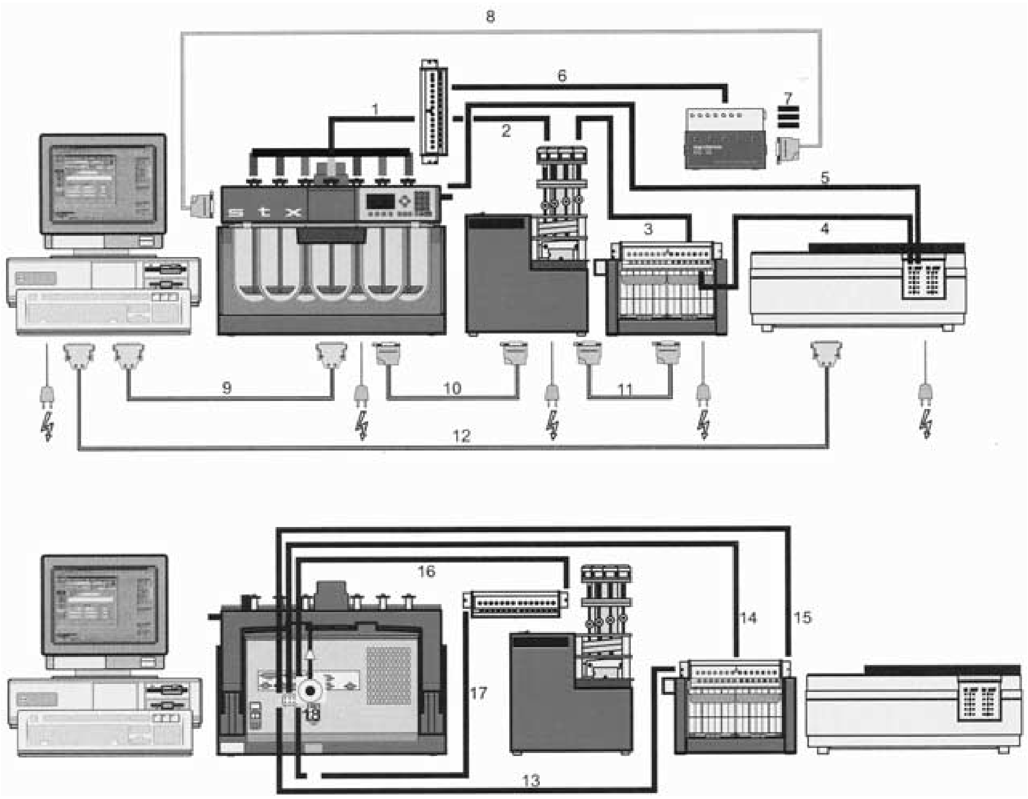

*Figure 5. AT 7*smart *On/Off-line System with the Media Selector for pH change.*

- Bottom vales and efficient cleaning system guaranteeing no carry over from test to test.
- Very high accuracy and reproducibility.
- All steps done simultaneously in all seven vessels giving maximum speed and high throughput.
- Autocompliance™ Concept automatically to adjust paddle height, wobble, sampling position, centring and sealing the unit to limit loss on evaporation to $<0.5\%/24\,h$.
- Flexible concept to allow customized solutions.

WinSOTAX controls the system and contains the methods with which all parameters are specified for the tests. A batch run of methods is programmed. With the start of the batch run, WinSOTAX loads the specified method and executes the test starting with the media selection and preparation, the supply of tablets, and the test execution with sampling, the filter changes and the sample evaluation.

The AT 70*smart* is equipped with a very efficient cleaning system, with two rotating cleaning heads that spray cleaning solution at 4 bar in all angles of the system. This efficiently prevents any carry over and cross-contamination from test to test. The cleaning consists of the following steps.

- Emptying of the vessels after each test run through the bottom valve system allowing one to remove capsule residues and even small sinkers or the pellet device.
- Multiple rinsing of the vessels and tubing with 4-bar jet cleaning. This is done before the test with the

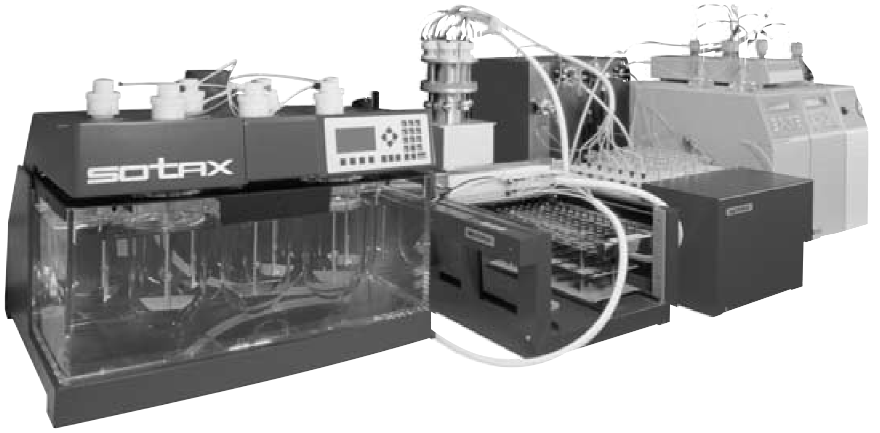

*Figure 6. AT 7*smart *HPLC On-line System.*

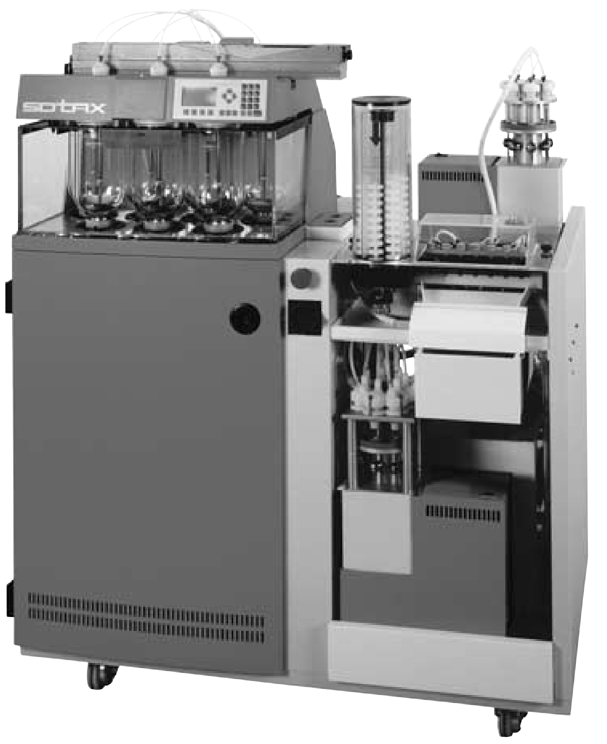

*Figure 7. AT 70*smart.

selected media and after the test with de-ionized water.
- A final purge with air to remove any remaining liquid. After this stage, the whole system is practically liquid free and new tests can be started.

The SOTAX AT 70*smart* is a very flexible system permitting different test procedures with the following operations.

- Solvent addition and/or full change with up to eight different solvents.
- Use of concentrates and modified media (SDS/SLS).
- Media replacement.
- Standard monitoring.
- Pellet testing.
- Tests with sinkers.

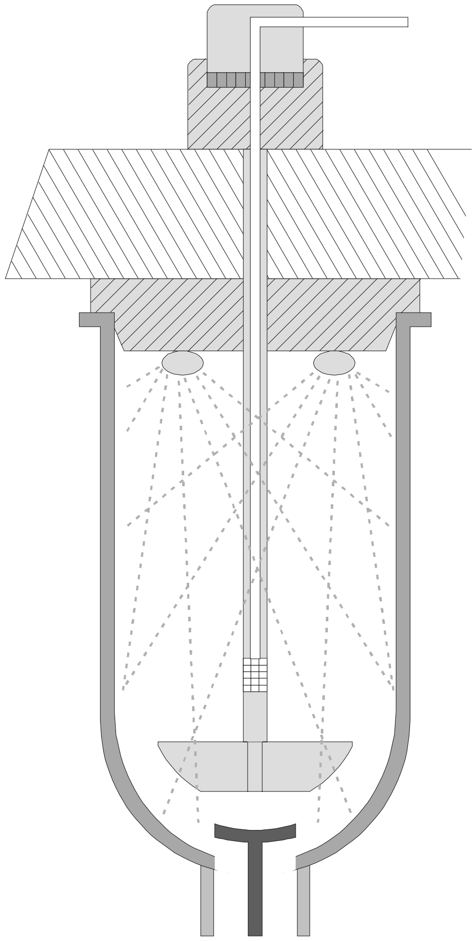

*Figure 8. Vessel cleaning.*

Tests with baskets require additionally the Basket-Station BS 60. Up to 10 USP 1 tests can be loaded. Several design features prevent any cross-contamination on the change of the basket. The holding systems for fresh and tested (contaminated) baskets are not identical. At the test end, baskets are precleaned and during lift up of the stirrer unit shock stops force drops to fall into the vessel. The baskets are then moved out of the tester and stored in a container and the next six baskets are transferred.

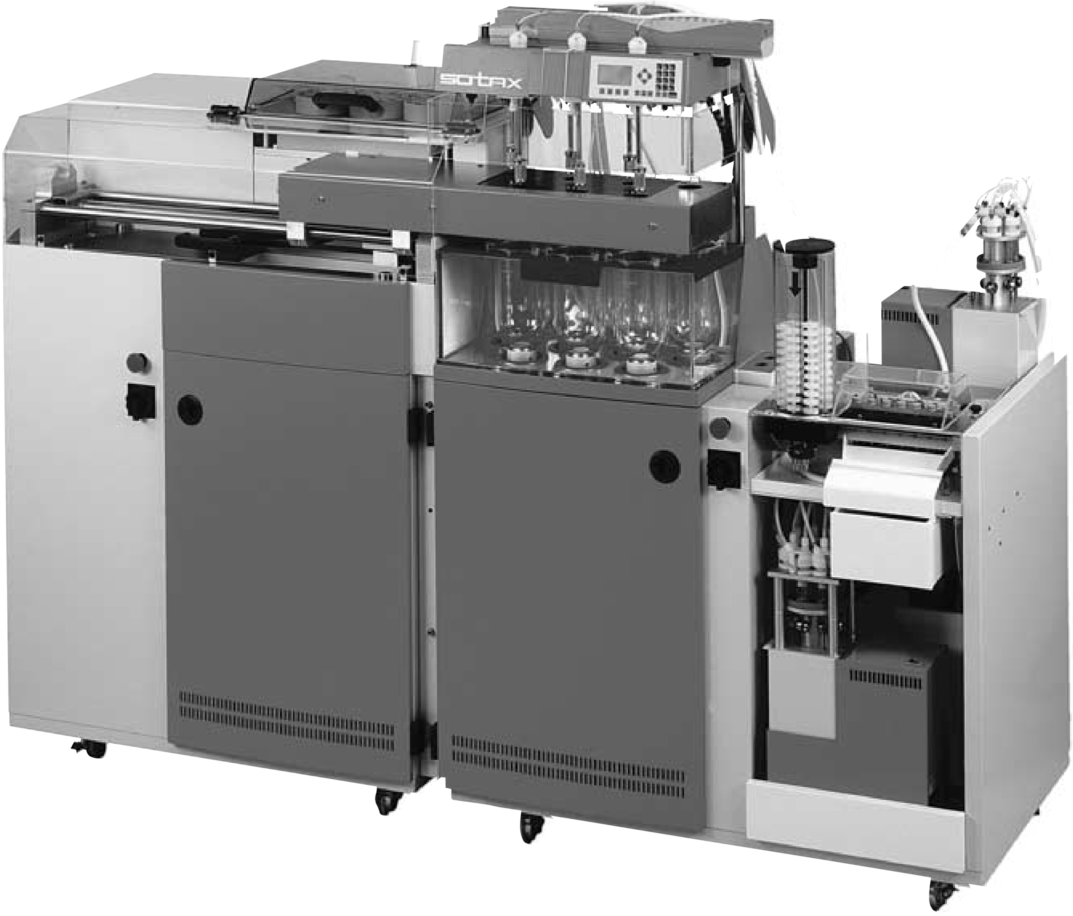

*Figure 9. AT 70*smart *with the Basket Station BS 60.*

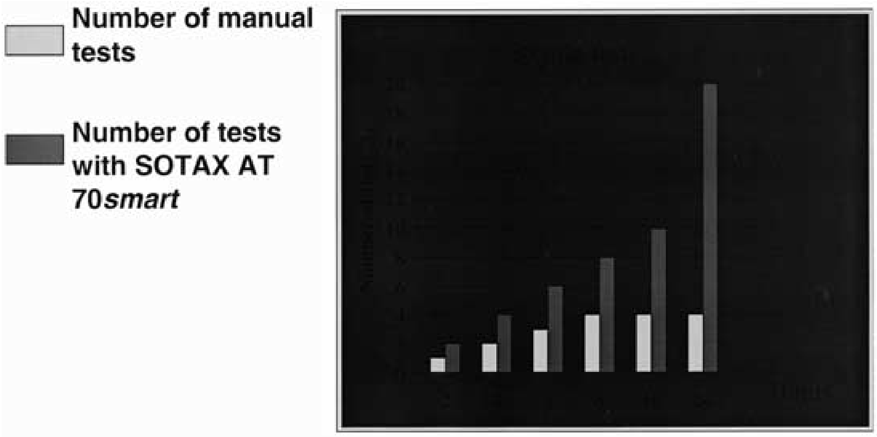

*Figure 10. Capacity increase with AT 70*smart.

The testing capacity by using the SOTAX fully automated system AT 70*smart* is increased considerably: in 24 h up to 20 tests can be realized against only four manual tests.

### Automation of USP 4 tests

SOTAX developed together with Ciba-Geigy and Hoechst this type of dissolution test, which often results in a better *in-vivo/in-vitro* correlation than with the USP 1 and 2 methods. As the USP 4 method is more laborious than the USP 1 and 2 methods, SOTAX developed a stand-alone unit CE 7*smart* already equipped with automated features such as test preparation, solvent change and system cleaning. In an offline configuration, a splitter is used to reduce the sample volume. For medium-change procedures, the media selector is used. The WinSOTAX software operates the system and executes all necessary calculations.

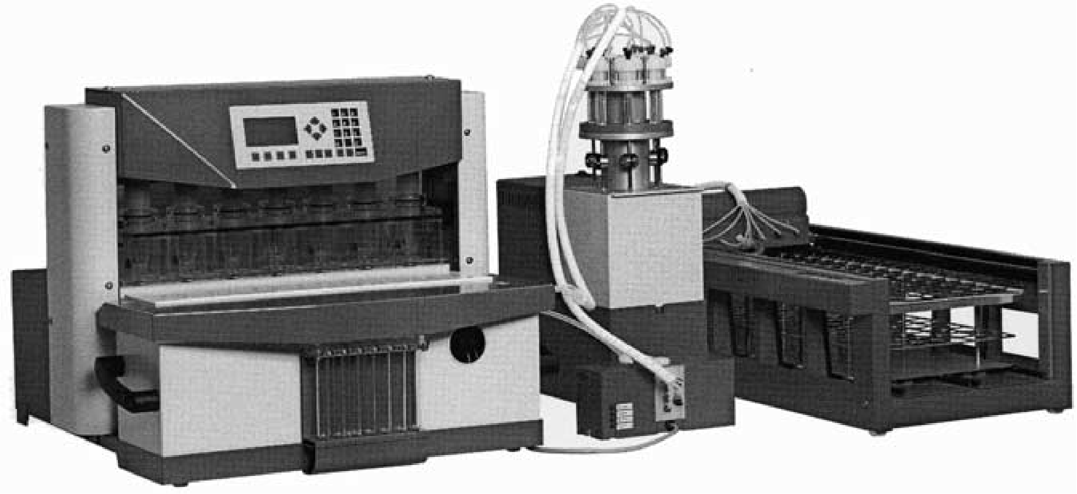

*Figure 11. CE 7*smart *Off-line System.*

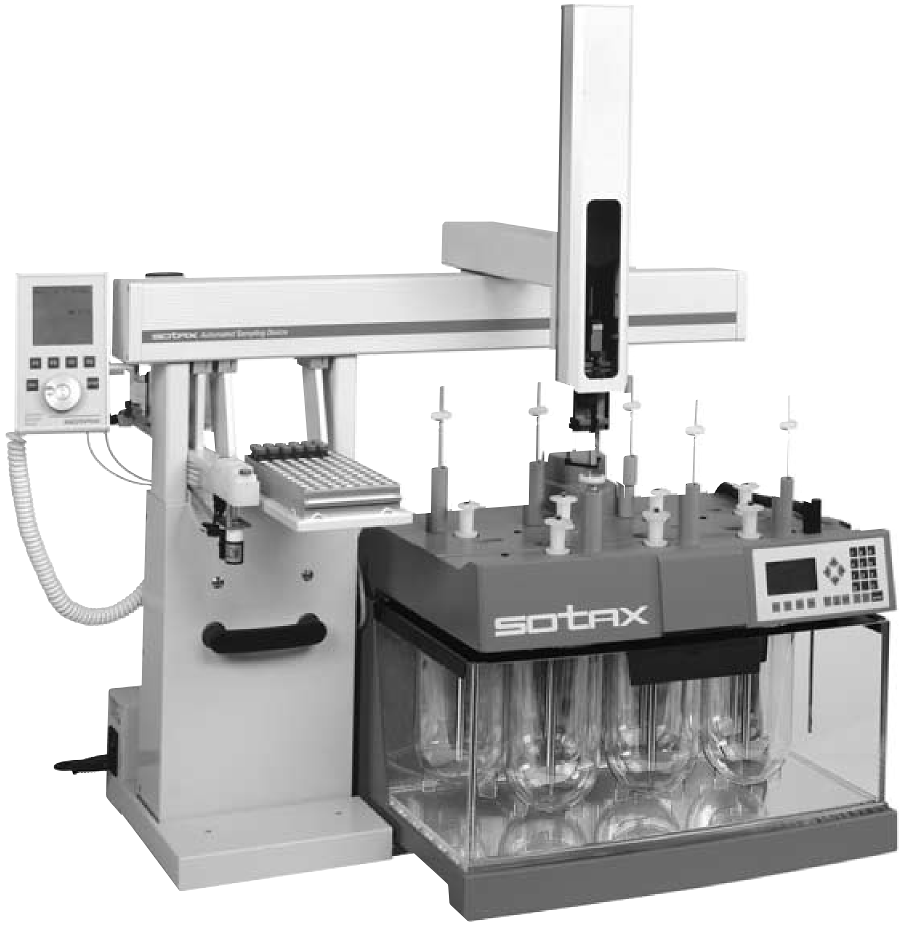

*Figure 12. AT 7*smart *with the Automatic Sampling Device.*

**Automation of TTS-patches tests**

There are two methods for testing of patches:

- USP 5 method (paddle over disc); and
- USP 6 method (TTS-cylinder).

Up to now, no automation concept for the USP 5 method has been developed. In Europe, the USP 6 method is more common. In the automation of USP 6, sampling is the key issue because in most cases patches are extended release preparations, e.g. hormones. Such patches are often analysed over 96 h with only a few samples taken (e.g. three sampling points). Because of the test length, sampling can occur during the night or at weekends and this causes some organizational problems. Therefore, automation of sampling is very important while automatically changing the TTS-cylinders has not been an issue so far. As the concentration of active substance is usually very low (μg level), the automation of the sampling needs special consideration and long tubes lead to adsorption. Sampling is realized with the Automatic Sampling Device (ASD) an X–Y–Z robotic system. The robotic arm equipped with a syringe takes the samples, which can be filtered and filled, directly into the vials. These samples can be kept cool with a Pelletier element while awaiting for further analysis.

### Software

The software for an automated system is considered even more important than the hardware. Most instrument suppliers have developed sophisticated software solutions. Since the introduction of 21 CFR Part 11 with very strict regulations about development documentation, change controls and data security, most suppliers are in the process of renewing their software. Fortunately, WinSOTAX was developed from 1997 onwards and 21 CFR Part 11 was taken in consideration once it was issued. It includes audit trails and an electronic signature. WinSOTAX makes it possible to realize different automation solutions and it controls all SOTAX dissolution systems. It is constantly extended with new features and covers all variations of dissolution testing. Besides the 21 CFR Part 11 requirements, WinSOTAX allows one to perform many different test procedures, customized reporting including statistical analysis, tabular data and graphs, as well as connecting to networks and export functions. It also offers the possibility for standard monitoring, different standard calculations, multicomponent analysis and cell grouping.

### Validation

Today validations of the systems have become the top priority of the pharmaceutical industry laboratory. The SOTAX Autocompliance™ concept design used for dissolution instruments automatically adjusts paddle height, the sampling position, centring and, with short shafts, eliminates wobble. This reduces the required time for validation considerably. SOTAX has worked together with leading pharmaceutical firms to create validation documents including all SOPs (standard operating procedure) and test protocols, which are available in different languages.

### Conclusion and outlook

The development and use of automated dissolution test systems helps one to cope with the increased number of tests. The required documentation has grown even faster and occupies a very high amount of work time for laboratory staff. Automation enables laboratories to be more efficient and improve the accuracy of their data. Some available automation concepts are very flexible, easy to validate and can be used during 24 h non-stop. Finally, the high gain in productivity gives a very short return on investment. Therefore, automation is expected to gain a higher share while manual systems are replaced or used only for long tests or for minor products.

